# Current Situation and Key Parameters for Improving Wheat Quality in China

**DOI:** 10.3389/fpls.2021.638525

**Published:** 2021-02-15

**Authors:** Mingming Ma, Yingchun Li, Cheng Xue, Wei Xiong, Zhengping Peng, Xue Han, Hui Ju, Yong He

**Affiliations:** ^1^Institute of Environment and Sustainable Development in Agriculture, Chinese Academy of Agricultural Sciences, Beijing, China; ^2^College of Resources and Environmental Science, Agricultural University of Hebei, Baoding, China; ^3^International Maize and Wheat Improvement Center (CIMMYT), Texcoco, Mexico

**Keywords:** wheat, quality traits, variation, correlation, cultivar, year, location

## Abstract

Processing quality of winter-wheat is affected by genotype, environmental conditions, and crop husbandry practices. In the present study, a data set of 17 quality-related traits for 211 main winter-wheat varieties in China during 2006 to 2018 was extracted from China Wheat Quality Report. Analysis was carried out to evaluate the quality status and variations, to reveal correlation between quality-related traits, as well as to identify key influencing factors. Results indicated that the quality indicators of medium-gluten or medium-strong-gluten wheat varieties were acceptable, whereas those of weak- and strong-gluten wheat varieties were far below national standard, especially hardness index (HI), crude protein content (CPC), wet gluten content (WG), and water absorption for weak-gluten wheat and sedimentation value (SV), stability time (ST), and stretch area (SA) for strong-gluten wheat, respectively. Correlation analysis showed that WA, WG, development time, HI, CPC, falling number, ST, and tractility directly affected the overall quality of winter-wheat. CPC, SV, and WG in medium-gluten wheat had no significant correlation with the processing quality of noodles score, whereas gluten index significantly correlated with noodle score (*P* < 0.001). This implied that protein quality might play a more important role than protein quantity in determining medium-gluten wheat quality. Furthermore, analysis of variance showed that genetic characteristics (cultivars) had significant influences on the restriction indexes (SV, ST, and SA) of strong-gluten wheat, whereas genetic characteristics, environment conditions, and crop growing practices (cultivars, locations, and years) significantly affected the restriction indexes (HI, CPC, WG, and WA) of weak-gluten wheat. The results suggest that improvement of Chinese strong-gluten wheat should mainly focus on cultivating new varieties. As to weak-gluten wheat, cultivation and husbandry practices should be paid more attention to limit undesired high grain protein content.

## Introduction

As the second staple crop in the world, wheat (*Triticum aestivum* L.) provides approximately 20% of protein and 20–40% of minerals for human nutrition globally (Food and Agriculture Organization of the United Nations [FAO], 2019). Besides, wheat supplies 20% of the calories consumed worldwide (Reynolds et al., 2012) and occupies approximately 25% of the global cereal production area (Punia et al., 2019). By 2018, cultivated area of winter-wheat accounts for 93.7% of the total area of wheat in China, and the production accounts for 95.1% (Ministry of Agriculture and Rural Affairs of PRC, 2020). In recent years, with the improvement of people’s living standards, the focus of China’s wheat production has gradually shifted from the pursuit of high grain yield to high processing quality. The quality of winter-wheat is classified into several categories (such as grain quality, flour quality, and dough quality), and they are determined by different quality indexes. For example, crude protein content (CPC), wet gluten content, gluten index (GI), development time (DT), and stretch area (SA) are some of the key factors in evaluating the quality of wheat-based food products. Noodles are one of the Chinese people’s favorite foods and play an important role in three meals a day. Therefore, their impacting factors of quality traits are needed to be explored in order to improve wheat quality.

Quality of wheat grain is jointly affected by genotypes, environmental conditions, and husbandry practices (Hellemans et al., 2018; Zörb et al., 2018). Cultivation measures, genetics, and milling and processing conditions were major factors affecting the quality of flour and the corresponding products (Kweon et al., 2011). Wheat quality was affected to some extent by the climatic conditions during irrigation, and the correlation coefficient could be positive, negative, or close to zero, depending on the temperature and water input at this stage (Rharrabti et al., 2003a,b). The sedimentation value (SV) of wheat under drought stress was lower than that under normal irrigation conditions (Magallanes-López et al., 2017). Environmental conditions also affected grain ash content (AC), which was increased under high transpiration conditions (Araus et al., 1998). Protein quality was mainly affected by wheat genotypes, whereas no obvious differences were found across production years (Torbica et al., 2016). During dough development, gluten quantity was the main factor determining consistency of dough (Fu et al., 2017), and the genotype had a significant effect on the GI (Šekularac et al., 2018). Protein and gluten properties, in particular, significantly impacted dough strength measurements. Cultivars displaying stronger gluten strengths may result in dough with better dough-handling properties (Tozatti et al., 2020). Falling number (FN) and protein concentration were highly influenced by environment, whereas for SV, hardness, water absorption (WA), and loaf volume genotypes accounted for more than 60% of total variation. Strong relations exist among protein concentration, SV, and loaf volume (Laidig et al., 2017). Moreover, it can be observed that the quality of different wheat cultivars, or even the same wheat cultivar, might vary significantly across growing regions and years (Mohan and Gupta, 2011).

Consistent emphasis on high grain yield and the lack of germplasm resources had led China to its wheat varieties concentrated mainly on medium-gluten and medium-strong- gluten wheat (Hu et al., 2017). Although grain protein content of wheat in China was comparable to that in Europe and the United States, dissatisfactory protein quality (composition of protein components and their subunits) caused weak-gluten strength, poor rheological properties of dough, low SV, and inferior processing quality (especially for strong-gluten and weak-gluten wheat). In addition, most of the current research focused only on the analysis of wheat quality in a single region (Rozbicki et al., 2015; da Silveira et al., 2020). There has been no systematic analysis and comprehensive comparison study on wheat quality in China. Even for evaluations of wheat quality, they were predominately based on one or several limited indicators (especially wheat grain protein content as the core evaluation indicator) (Delcour et al., 2012).

Studies on independent as well as interacted effects of cultivars, growing locations, and years on winter-wheat quality in China were limited. Nevertheless, such information is essential for breeding, regional planting arrangement, and large-scale production of high-quality wheat. Quality properties that are predominantly determined by genotypes could be improved through crop breeding, and those significantly affected by external environmental conditions might be adjusted and optimized via husbandry management. In this study, data were extracted from the China Wheat Quality Report published by the Ministry of Agriculture of the People’s Republic of China. A variety of statistical analysis methods were carried out to screen and analyze a total of 17 quality traits for the 211 winter-wheat cultivars during 2006–2018 in six growing regions in China. The main objectives of this study were (1) to evaluate current situation of winter-wheat quality in China, (2) to identify key parameters limiting the quality improvement, and (3) to analyze the impacts of different cultivars, locations, and years on quality traits.

## Materials and Methods

### Data Source

**FIGURE 1:**
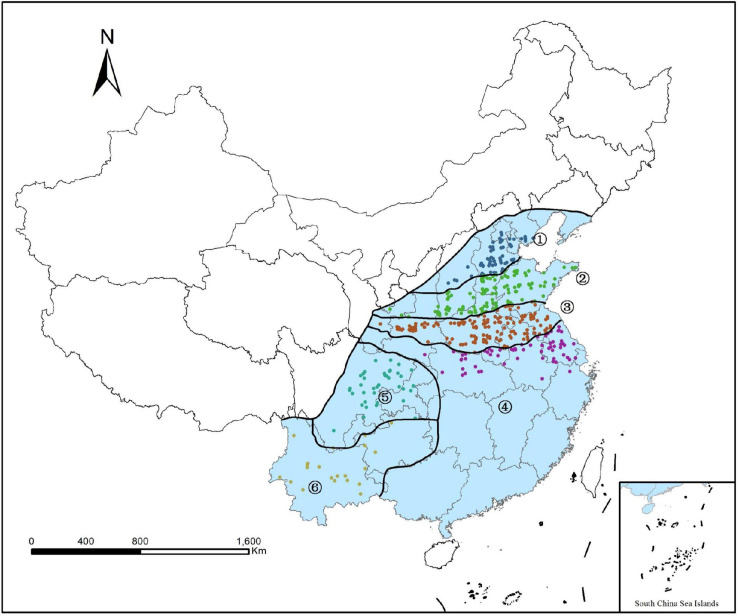
Distributions of sampling areas and six growing regions of winter-wheat in China (MARA, 2016).

We selected wheat cultivars data on four quality-related parameters from the 2006–2018 China Wheat Quality Report published by the Ministry of Agriculture of the People’s Republic of China, and box chart method was performed to eliminate outliers. In total, a set of 1,055 winter-wheat quality data for 211 cultivars was collected. According to the classification guide of wheat quality regions in China (He et al., 2002), winter-wheat growing areas were divided into six regions (Figure 1), including North strong-gluten wheat region (1), North Huanghuai strong- and medium-strong-gluten wheat region (2), South Huanghuai medium-gluten wheat region (3), the medium-weak-gluten wheat region in the middle and lower Reaches of the Yangtze River (4), Sichuan Basin medium-weak-gluten wheat region (5), and Yungui Plateau wheat region (6).

### Statistical Analysis

#### Standard-Reaching Rates and Variation Analysis

According to the China National Standard GB/T 17320-2013 (AQSIQ, 2013), eight quality parameters (Table 1), including hardness index (HI), CPC, SV, wet gluten content (WG), WA, stability time (ST), max resistance (MAXR), and SA were analyzed for the standard-reaching rates (SRs) of winter-wheat.

**TABLE 1:** National standard for wheat quality in China.

**Index**	**Strong-gluten**	**Medium-strong-gluten**	**Medium-gluten**	**Weak-gluten**
Hardness index (HI)	≥60	≥60	≥50	<50
Crude protein content (CPC)	≥14	≥13	≥12.5	<12.5
Sedimentation value (SV)	≥40	≥35	≥30	<30
Wet gluten content (WG)	≥30	≥28	≥26	<26
Water absorption (WA)	≥60	≥58	≥56	<56
Stability time (ST)	≥8	≥6	≥3	<3.0
Max resistance (MAXR)	≥350	≥300	≥200	–
Stretch area (SA)	≥90	≥65	50	–

Descriptive statistics were applied to evaluate the variability of examined factors: the means, minimum, maximum, and standard deviations. Coefficients of variation (CVs) were also calculated (variation analysis).

#### Correlation Analysis

Pearson correlation analysis was performed on 17 quality traits of winter-wheat using R 3.6.2 (R package corrplot).

#### Multifactor Analysis of Variance

Multifactor analysis of variance was conducted using R 3.6.2 to evaluate the effects of the examined factors and their interactions on grain, flour, and dough quality. In the homogeneity of variance and normal distribution using R 3.6.2 (R package car), the results show that the data are normally distributed, and the variance is homogeneous.

## Results

### The Standard-Reaching Rates of Winter-Wheat Quality Traits

SRs of different grades of winter-wheat were analyzed according to the national standard GB/T 17320-2013 (Table 2). In terms of HI, the SRs of the medium-gluten wheat reached 91%, but that of weak-gluten wheat was only 9%. The SRs of CPC for medium-, medium- strong-, strong-, and weak-gluten wheat were 87, 79, 54, and 13%, respectively. In terms of the SV, the SRs of medium-, medium- strong-, weak-, and strong-gluten wheat were 54, 28, 46, and only 11%, respectively. For WG, 90% of the medium-gluten, 79% of the medium-strong-gluten, 61% of the strong-gluten and only 10% of the weak-gluten wheat samples met the corresponding standards. The SRs of the medium-, medium- strong-, strong-, and weak-gluten wheat for WA were 77, 60, 41, and 23%, respectively. When it came to the ST, the SRs of the medium-, medium- strong-, weak-, and strong-gluten wheat were 69, 33, 31, and 24%, respectively. The SRs of MAXR for medium-, medium- strong-, and strong-gluten wheat were 93, 82, and 67%, respectively. Finally, the SRs of SA for the medium-, medium- strong-, and strong-gluten wheat were 90, 68, and 27%. The results showed that the SRs of medium-gluten and medium-strong-gluten winter-wheat were relatively higher compared with those of strong-gluten and weak-gluten wheat in China. Analysis of quality traits showed that for weak-gluten wheat, the SRs of HI, WG, CPC, and WA were relatively low, whereas for strong-gluten wheat, the SRs of SV, ST, and SA were relatively low.

**TABLE 2:** Analysis of quality traits reaching the national wheat quality standard.

**Indexes**	**Strong-gluten**	**Medium-strong-gluten**	**Medium-gluten**	**Weak-gluten**
Hardness index (HI)	78 (62–75)	78 (62–72)	91 (50–75)	9 (32–49)
Crude protein content (CPC) (%)	54 (14–18)	79 (14–18)	87 (13–19)	13 (9–12)
Sedimentation value (SV) (mL)	11 (40–52)	28 (35–44)	54 (30–66)	46 (5–25)
Wet gluten content (WG) (%)	61 (30–44)	79 (30–50)	90 (26–47)	10 (16–25)
Water absorption (WA) (%)	41 (60–70)	60 (58–71)	77 (56–71)	23 (51–54)
Stability time (ST) (min)	24 (8–20)	33 (6–35)	69 (3–54)	31 (1–3)
Max resistance (MAXR) (E.U)	67 (416–969)	82 (302–774)	93 (200–1,638)	–
Stretch area (SA) (cm^2^)	27 (90–215)	68 (68–215)	90 (32–239)	–

*Data outside the brackets indicate the percentages of the compliance rate, and data inside brackets represent the variation range of wheat samples that reached the national wheat quality standard. “–” represents data not included in the standard.*

### Analysis of Variation of Winter-Wheat Quality Traits

Based on the variation analysis of quality traits (Table 3), the CVs were listed as follows: ST > MAXR > SA > DT > GI > TRA > SV > FN > AC > WG> HI>CPC >WC> FER > WA > NS > TW. The CV of ST was the highest among all quality traits, reaching 79.3%. The CVs of MAXR, SA, DT, GI, TRA, and SV were 49.9, 48.4, 43.2, 28.4, 23.3, and 22.2%, respectively. The CVs of FN, AC, and WG were 16.5, 15.4, and 12.2%, respectively. The CVs of HI, CPC, WC, FER, WA, and NS were relatively low at 9.5, 8.9, 8.2, 6.4, 6.1, and 4.6%, respectively. The CV of TW was the smallest at only 2.9%.

**TABLE 3:** Variation of analysis wheat quality traits.

**Categories**	**Indexes**	**Minimum**	**Maximum**	**Average**	***SD***	**CV (%)**
Grain quality	Hardness index (HI)	32.0	75.0	63.7	6.0	9.5
	Test weight (TW) (g L^–1^)	703.0	854.0	795.8	22.9	2.9
	Water content (WC) (%)	7.9	14.3	10.8	0.9	8.2
	Crude protein content (CPC) (%)	9.0	18.8	14.0	1.2	8.9
	Falling number (FN) (s)	73.0	529.0	360.8	59.4	16.5
Flour quality	Flour extraction rate (FER) (%)	11.5	77.3	67.9	4.3	6.4
	Sedimentation value (SV) (mL)	5.0	66.2	30.6	6.8	22.2
	Ash content (AC) (%)	0.3	0.9	0.5	0.1	15.4
	Wet gluten content (WG) (%)	16.4	50.0	30.9	3.8	12.2
	Gluten index (GI) (%)	0.0	100.0	68.7	19.5	28.4
Dough quality	Water absorption (WA) (%)	51.0	71.0	58.9	3.6	6.1
	Development time (DT) (min)	0.7	11.2	3.3	1.4	43.2
	Stability time (ST) (min)	0.7	54.2	5.3	4.2	79.3
	Stretch area (SA) (cm^2^)	4.0	239.0	87.4	42.3	48.4
	Tractility (TRA) (mm)	59.0	665.0	156.3	36.5	23.3
	Max resistance (MAXR) (E.U)	30.0	1, 638.0	424.8	212.0	49.9
Processing quality	Noodles score (NS)	70.0	91.0	81.3	3.7	4.6

*SD, standard deviation; CV, coefficient of variation.*

### Correlation Analysis of Winter-Wheat Quality Traits

**FIGURE 2:**
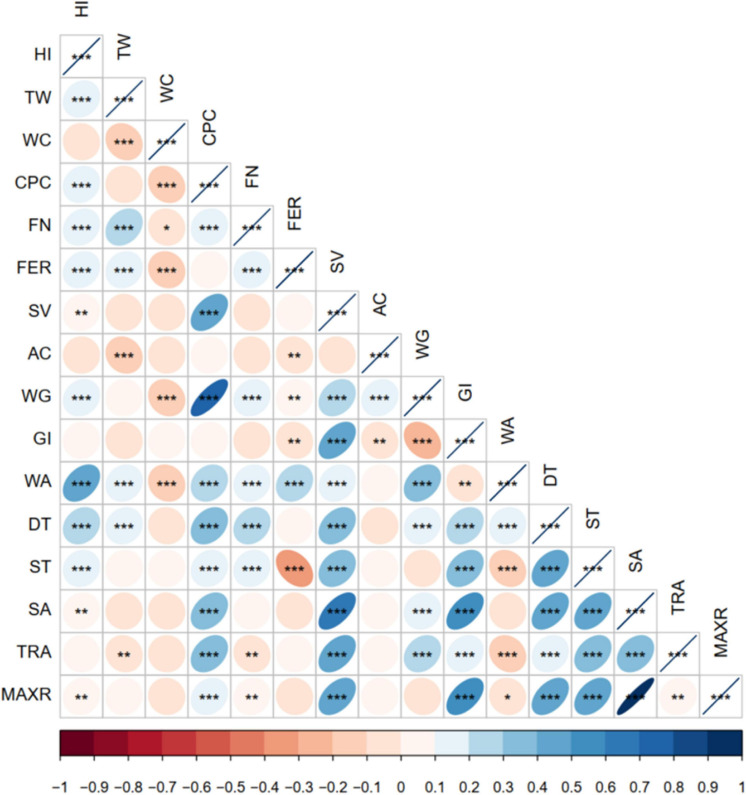
Correlation analysis of all winter-wheat quality traits. Significant correlation at *0.05, **0.01, and ***0.001 levels (bilateral), respectively.

The correlations of winter-wheat quality-related parameters are summarized in Figure 2. On the whole, wheat quality indicators were closely correlated, especially WG and CPC, SA and SV, SA and GI, MAXR, and GI. In terms of grain quality, only FN was significantly correlated with HI, test weight (TW), water content (WC), and CPC. This indicated that FN could be the most important indicator for evaluating grain quality, followed by HI, TW, and WC. In terms of flour quality, WG was correlated with the other quality traits, which involved flour extraction rate (FER), SV, AC, and WG, and GI was correlated with the other five quality traits (FER, SV, AC, WG, and GI). This suggested that WG and GI were the most important indicators of flour quality. Moreover, with regard to dough quality, DT, ST, and tractility (TRA) were significantly correlated with other dough quality, indicating that DT and ST could be the core trait of dough quality.

On the whole, WA was significantly correlated with 13 other traits of winter-wheat quality. WG and DT were significantly correlated with 12 other traits of winter-wheat quality. HI, CPC, FN, ST, and TRA were significantly correlated with 11 other traits. These results indicated that WA, WG, DT, HI, CPC, FN, ST, and TRA directly affected the overall quality of winter-wheat.

**FIGURE 3:**
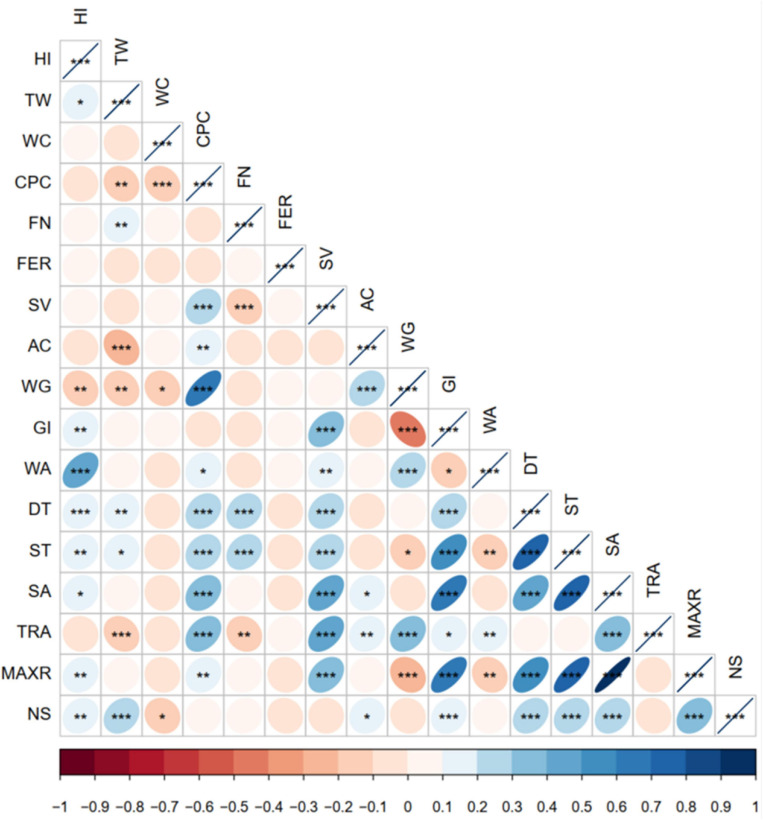
Correlation analysis of wheat samples meeting the standard of medium-gluten and noodle scores. Significant correlation at *0.05, **0.01, and ***0.001 levels (bilateral), respectively.

The correlation analysis of wheat samples meeting the standard of medium-gluten and noodle scores is presented in Figure 3. There was a close correlation between indicators of wheat samples achieving medium-gluten SRs (GB/T 17320-2013), especially the relationship between WG and CPC, ST and DT, ST and GI, SA and GI, SA and ST, MAXR and GI, MAXR and DT, MAXR and ST, and MAXR and SA. Noodles score (NS) has a significant correlation with HI, TW, WC, AC, GI, DT, ST, SA, and MAXR.

### Variance Analysis of Winter-Wheat Quality Traits

According to the analysis of variance, the effects of growing locations, cultivars, and years varied across different traits (Table 4). Although interaction effects existed, winter-wheat quality parameters were mainly affected by single factor effect (i.e., locations, cultivars, or years). Specifically, HI, TW and CPC, FER, and SV were significantly affected by location and cultivars, but were not influenced by years, because these index interactions between locations and years significantly affected HI and FER. WC, FN, GI, WA, and TRA were significantly affected by locations, years, and cultivars. SA and MAXR were only significantly affected by cultivars. AC was only significantly affected by growing locations. For ST and DT, no significant influences were detected between locations, cultivars, years, and their interactions.

**TABLE 4:** Variance analysis of winter-wheat quality traits.

**Categories**	**Traits**	**Location (L)**	**Cultivar (C)**	**Year (Y)**	L*C	L*Y	C*Y	L*C*Y
Grain quality	Hardness index (HI)	**	**	ns	ns	*	*	ns
	Test weight (TW)	**	**	ns	ns	ns	ns	ns
	Water content (WC)	**	**	**	ns	**	*	ns
	Crude protein content (CPC)	**	**	ns	ns	ns	ns	ns
	Falling number (FN)	**	**	**	**	**	*	ns
Flour quality	Flour extraction rate (FER)	**	**	ns	ns	**	**	ns
	Sedimentation value (SV)	**	**	ns	ns	ns	ns	ns
	Ash content (AC)	**	ns	ns	ns	ns	ns	ns
	Wet gluten content (WG)	**	**	**	ns	ns	ns	ns
	Gluten index (GI)	**	**	**	**	ns	ns	ns
Dough quality	Water absorption (WA)	**	**	**	**	**	*	ns
	Development time (DT)	ns	ns	ns	ns	ns	ns	ns
	Stability time (ST)	ns	ns	ns	ns	ns	ns	ns
	Stretch area (SA)	ns	**	ns	ns	ns	ns	ns
	Tractility (TRA)	**	**	**	ns	**	**	ns
	Max resistance (MAXR)	ns	**	ns	ns	ns	ns	ns

*Significant at P < 0.05 level. **Significant at P < 0.01 level. ns, not significant.

## Discussion

### Restricting Parameters for Strong-Gluten Wheat and Weak-Gluten Wheat

In recent years, China has made remarkable achievements in increasing wheat yield and total production. At the same time, with the increased income of residents, their demand for wheat quality is also upgrading. In general, the quality of medium- and medium-strong-gluten wheat in China is relatively high and can basically meet the market quality requirements. However, the strong-gluten wheat used for making bread and the weak-gluten wheat for cake are far from meeting the market demand, making it less competitive compared with high-quality agricultural products from other countries (Deliberali et al., 2010). As a result, a significant quantity of high-quality strong- and weak-gluten wheat was imported each year.

Currently, many researches focus on identifying how the wheat quality could meet the strong-gluten or weak-gluten standard more quickly and accurately, so as to obtain optimal traits for wheat breeding. Compared with other testing techniques, rheometry is considered as a better technique to predict final product quality (Dobraszczyk and Morgenstern, 2003; Hrušková et al., 2006). Based on China National Standard (GB/T 17320-2013), the SRs of Chinese wheat quality indicators were different among strong-, medium-, medium-, and weak-gluten. SRs of HI, WG, MAXR, and WA were all greater than 90% (Table 2) in medium-gluten wheat, indicating that the quality of medium-gluten wheat was relatively high in China. These results suggested that HI, WG, MAXR, and WA were no longer limiting factors in quality improvement of medium-gluten wheat in China. However, the SRs of weak-gluten wheat were relatively low in China, especially for HI (9%), CPC (13%), WG (10%), and WA (23%) (Table 2). However, the rheological index is time-consuming and laborious, which is not conducive to quick grasp. Our results indicate that HI, CPC, WG, and WA had become major factors affecting the development of weak-gluten wheat in China. Therefore, it could be applied to determine whether the wheat meets the standard of weak-gluten wheat by determining the quality of HI, CPC, WG, and WA. The latest research also suggests that WA, CPC, and WG should be the main factors in the quality selection of weak-gluten wheat (Hu et al., 2020). For weak-gluten wheat in China, WG and CPC are not insufficient, but even high. Hu et al. (2020) think field management is difficult to take into account the quality, leading to the high CPC and WG. Therefore, the extreme value in the national standard should be relaxed appropriately.

The overall compliance rate of strong-gluten wheat was higher than that of weak-gluten wheat, but strong-gluten wheat quality in China still needs to be improved. This study found that the improvement of strong-gluten wheat was limited by SV, ST, and SA (Table 2). Regarding ST and SA, the low SRs in association with extremely high CV (Tables 2, 3) implied that the large variability of ST (Zhao et al., 2019) and SA should be a key reason responsible for the low SRs of strong-gluten wheat in China. Consequently, special attention should be paid to select strong-gluten wheat cultivars with long dough ST and strong tensile properties. Furthermore, providing a suitable growth environment is essential to ensure the quality stability.

### Correlation Among Quality Indexes of Winter-Wheat

Multiple quality parameters for winter-wheat that are closely related to each other made it difficult to evaluate wheat quality. Based on the correlation analysis of 13 years’ data of six major wheat regions in China, CPC and WG are significantly correlated (Figure 2), and this result is consistent with the that of Rozbicki et al. (2015). Previous study showed that gliadin and glutenin are the main components of wet gluten (Shewry et al., 2000), and gluten comprises some 75% protein on a dry weight basis, with most of the remainder being starch and lipids (Shewry et al., 2002). This may be the main reason for the significant correlation between CPC and WG. In addition, we found that GI, MAXR, and SA were all significantly correlated. On the one hand, the high GI value indicated that the quality of glutenin and gliadin was high, and high-quality glutenin provides the tensile resistance of the dough, whereas high-quality gliadin provides the adhesion required for dough fluidity and ductility. However, there was no significant correlation between WG and MAXR. This result indicates that the composition and structure of protein may have a more important effect on quality than protein quantity, which is similar to the result of Xue et al. (2016b). On the other hand, both SA and MAXR are extensibility indexes of dough. SA indicates dough strength: higher value of SA reflects greater strength of dough. There is a significant positive correlation between SA and MAXR.

The correlation between the quality indicators of medium-gluten wheat and NS showed that NS has no significant correlation with quantitative parameters of proteins (CPC, SV, and WG), but significant correlation (*P* < 0.001) with protein composition index (GI) (Figure 3). Previous research found that the ratio of gliadin to glutenin and their subunit composition, especially the composition and proportion of high-molecular-weight glutenin subunits, had a significant effect on the baking quality of wheat (Xue et al., 2016a). This may be one of the reasons that the correlation between noodle quality and protein content indexes of medium-gluten wheat was not significant, but highly correlated with protein composition index.

### Influencing Factors of Winter-Wheat Quality in China

Genotype and environmental conditions are internal and external factors affecting the quality of winter-wheat. This study selected three influencing factors including growing locations, years, and cultivars. Growing locations represented husbandry practices and the spatial characteristics of soil and climatic conditions. Wheat cultivars represented genetic characteristics of the winter-wheat. Growing years mainly reflects the interannual climate conditions. Our results indicated that SV, ST, and SA were the main factors limiting the development of strong-gluten wheat (Table 2). The CV of ST (79.3%) was the largest (Table 3); the high CV of ST may be due to the fact that there was no effect of locations, cultivars, and years on ST, which was consistent with the study of Baric et al. (2004). However, cultivars had significant effects on SV and SA (*P* < 0.01), and especially SV had a high genetic stability (Moonen et al., 1982; Yong et al., 2004). Therefore, there is high potential for future development of strong-gluten wheat in China through breeding new varieties with excellent genes.

On the contrary, the quality of weak-gluten wheat was mainly restricted by HI, CPC, WG, and WA (Table 2). The results in Table 4 showed that HI, CPC, WG, and WA were significantly affected by locations and cultivars (*P* < 0.01). However, Rozbicki et al. (2015) found that CPC and WG were more significantly affected by environment than genotypes (varieties). Therefore, the core influencing factors of CPC and WG in different countries are not consistent. The interaction among wheat locations, varieties, and years had little effect on wheat quality, which was consistent with the results of Peterson et al. (1998). In addition, an important portion of the observed variability in quality was determined by the environment, and the nitrogen supply was the principal factor determining protein content and composition (Xue et al., 2019). The total protein content increases with higher supplies of nitrogen, and as the grain protein increases, the gliadin and glutenin contents and their ratio also increased (Triboi et al., 2000). As a consequence, breeding of wheat cultivars with low protein content and WG should be placed at the first priority in improving weak-gluten winter-wheat quality in China. It is suggested to strictly control the topdressing of wheat at the later stage and to ensure the irrigation amount before and after grain filling stage (Xue et al., 2016a).

## Conclusion

The winter-wheat quality status in China and their impact factors were analyzed in the present study. In general, the quality of medium-gluten or medium-strong-gluten wheat in China was relatively high, which can basically meet quality requirements. However, SRs of quality traits in strong- and weak-gluten wheat were not high, especially for SV, ST, and SA in strong-gluten varieties, as well as for HI, CPC, WG, and WA in weak-gluten winter-wheat. As a result, China imported a proportion of high-quality strong- and weak-gluten each year to satisfy market demand.

Medium-gluten wheat in the quantitative parameters of proteins (CPC, SV, WG) had no significant correlation on the processing quality of NS, but the composition parameter of proteins (GI) was very significant for noodle score, which indicates that protein composition is more important than protein content on determining the processing quality. The analysis of variance showed that genetic characteristics had significant influence on the restriction indexes of strong-gluten wheat (SV, ST, and SA), and genetic characteristics, ecological environment, and management measures had significant influence on the restriction indexes of weak-gluten wheat quality (HI, CPC, WG, and WA). Analysis on the influencing factors of different winter-wheat quality indicators is useful to accelerate variety selection and strengthen their environmental adaptation and ultimately improve winter-wheat quality in China.

## Data Availability Statement

The raw data supporting the conclusions of this article will be made available by the authors, without undue reservation, to any qualified researcher.

## Author Contributions

MM: data curation and writing—original draft. YL: conceptualization and writing—review and editing. CX: visualization and investigation. WX: method. ZP and YH: investigation. XH: formal analysis. HJ: project administration and supervision. All authors discussed the results as well as read and approved the final manuscript for publication.

## Conflict of Interest

The authors declare that the research was conducted in the absence of any commercial or financial relationships that could be construed as a potential conflict of interest.
